# Multiomics Studies Investigating Recurrent Pregnancy Loss: An Effective Tool for Mechanism Exploration

**DOI:** 10.3389/fimmu.2022.826198

**Published:** 2022-04-27

**Authors:** Jianan Li, Linlin Wang, Jinli Ding, Yanxiang Cheng, Lianghui Diao, Longfei Li, Yan Zhang, Tailang Yin

**Affiliations:** ^1^ Reproductive Medicine Center, Department of Obstetrics and Gynecology, Renmin Hospital of Wuhan University, Wuhan, China; ^2^ Shenzhen Key Laboratory of Reproductive Immunology for Peri-implantation, Shenzhen Zhongshan Institute for Reproduction and Genetics, Shenzhen Zhongshan Urology Hospital, Shenzhen, China; ^3^ Department of Clinical Laboratory, Renmin Hospital of Wuhan University, Wuhan, China

**Keywords:** omics, villus, decidua, blood, recurrent pregnancy loss

## Abstract

Patients with recurrent pregnancy loss (RPL) account for approximately 1%-5% of women aiming to achieve childbirth. Although studies have shown that RPL is associated with failure of endometrial decidualization, placental dysfunction, and immune microenvironment disorder at the maternal-fetal interface, the exact pathogenesis remains unknown. With the development of high-throughput technology, more studies have focused on the genomics, transcriptomics, proteomics and metabolomics of RPL, and new gene mutations and new biomarkers of RPL have been discovered, providing an opportunity to explore the pathogenesis of RPL from different biological processes. Bioinformatics analyses of these differentially expressed genes, proteins and metabolites also reflect the biological pathways involved in RPL, laying a foundation for further research. In this review, we summarize the findings of omics studies investigating decidual tissue, villous tissue and blood from patients with RPL and identify some possible limitations of current studies.

## Introduction

Recurrent pregnancy loss (RPL), defined as two or more consecutive clinically recognized spontaneous pregnancy losses before 20 weeks of gestation and includes embryonic or fetal loss, is a frequently occurring human infertility-related disease that affects 1-5% of parturients (1). Various factors have been proven to cause the occurrence and development of RPL, including chromosomal abnormalities, genital tract anomalies, immunological diseases, endocrine diseases, antiphospholipid syndrome, thrombophilic disorders and pathogen infections (2). Approximately 40-50% of cases remain unexplained, and the molecular mechanisms have not been fully explored. These cases are defined as unexplained recurrent pregnancy loss (URPL) (3). Although the diagnosis of RPL is relatively clear, the lack of standardized definitions, the uncertainties of its pathogenesis and the variable clinical manifestations still hamper progress in the treatment and prevention of RPL (4).

Omics studies generate a large amount of information concerning the biomarkers, molecular mechanisms and biological pathways involved in complex diseases (5). More recently, due to advances in the development and optimization of high-throughput techniques, numerous studies have applied omics approaches to the study of RPL (6–8). In this review, we summarize the results of omics-based studies conducted using human samples (decidua, villus, and blood) to explain the pathophysiological processes of RPL. The keywords in the included references were omics, omics technology, genomics, epigenomics, transcriptomics, proteomics, metabolomics, recurrent miscarriage, recurrent pregnancy loss, trophoblast, villi, decidua, and blood. We also attempted to perform an integrative analysis of omics data to obtain a global depiction of the complex relationships within and between different biological layers in RPL.

## Overview of Omics

Omics technologies, including genomics, epigenomics, transcriptomics, proteomics (9, 10) and metabolomics (11), provide a holistic and integrative approach toward the study of biological systems (12). Through genomics technology, we can collectively characterize and quantitatively analyze all genes of an organism and then study the structure, function, location, and editing of the genome and their impact on the organism. In genomics, genotype arrays and next-generation sequencing are mostly used to obtain copy number variations, single nucleotide variations and small insertions or deletions (13, 14). Epigenomics refers to the study of all epigenetic modifications at the genome level, and epigenetic modifications are heritable. The most pivotal epigenetic modifications are DNA methylation, histone modifications and nucleosome remodeling. The current epigenetic studies investigating RPL mainly focus on DNA methylation (15). The main methods of DNA methylation analyses are bisulfite sequencing, array or bead hybridization, pyrosequencing, methylation-specific polymerase chain reaction (PCR), high -performance liquid chromatography-ultraviolet, and mass spectrometry (MS)-based approaches (16). The transcriptomics approach, including microarrays, bulk RNA sequencing (RNA-seq) and single-cell RNA-seq (scRNA-seq), is often used to study gene expression and regulation. Qualitative and quantitative information concerning mRNA and noncoding RNA is available due to these approaches (17). Proteomics is the systematic and holistic study of the types, structures and functions of proteins expressed in cells or tissues. Proteomics methods mainly include protein microarray, gel-based approaches [two-dimensional polyacrylamide gel electrophoresis (2-DE)], MS-based approaches (18), X-ray crystallography, nuclear magnetic resonance spectroscopy, SOMAmer-based technology, and quantitative techniques, including isotope-coded affinity tag labeling, stable isotope labeling with amino acids in cell culture and isobaric tags for relative and absolute quantitation (iTRAQ) (19, 20). However, most proteomics studies investigating RPL were performed using 2D-DIGE or quantitative techniques, such as iTRAQ combined with MS-based approaches. Metabolomics usually identifies and quantifies metabolites, such as amino acids, lipids, sugars and hormones, *via* nuclear magnetic resonance, gas chromatography-mass spectrometry and liquid chromatography-mass spectrometry. By exploring the relative relationship between metabolites and physiopathological changes, it is possible to find biomarkers for the diagnosis of disease. By studying and integrating data obtained using different omics approaches, knowledge of the underlying molecular interactions and associated longitudinal effects may be discovered and understood more deeply (21). In some studies investigating RPL, omics technologies were used in combination with bioinformatics analyses, and key genes and molecular pathways affecting RPL were identified. Upregulated or downregulated genes have been found to participate in a variety of biological processes important for embryonic/fetal development and fertility (22–24).

## Establishment and Maintenance of Early Pregnancy

**Figure 1:**
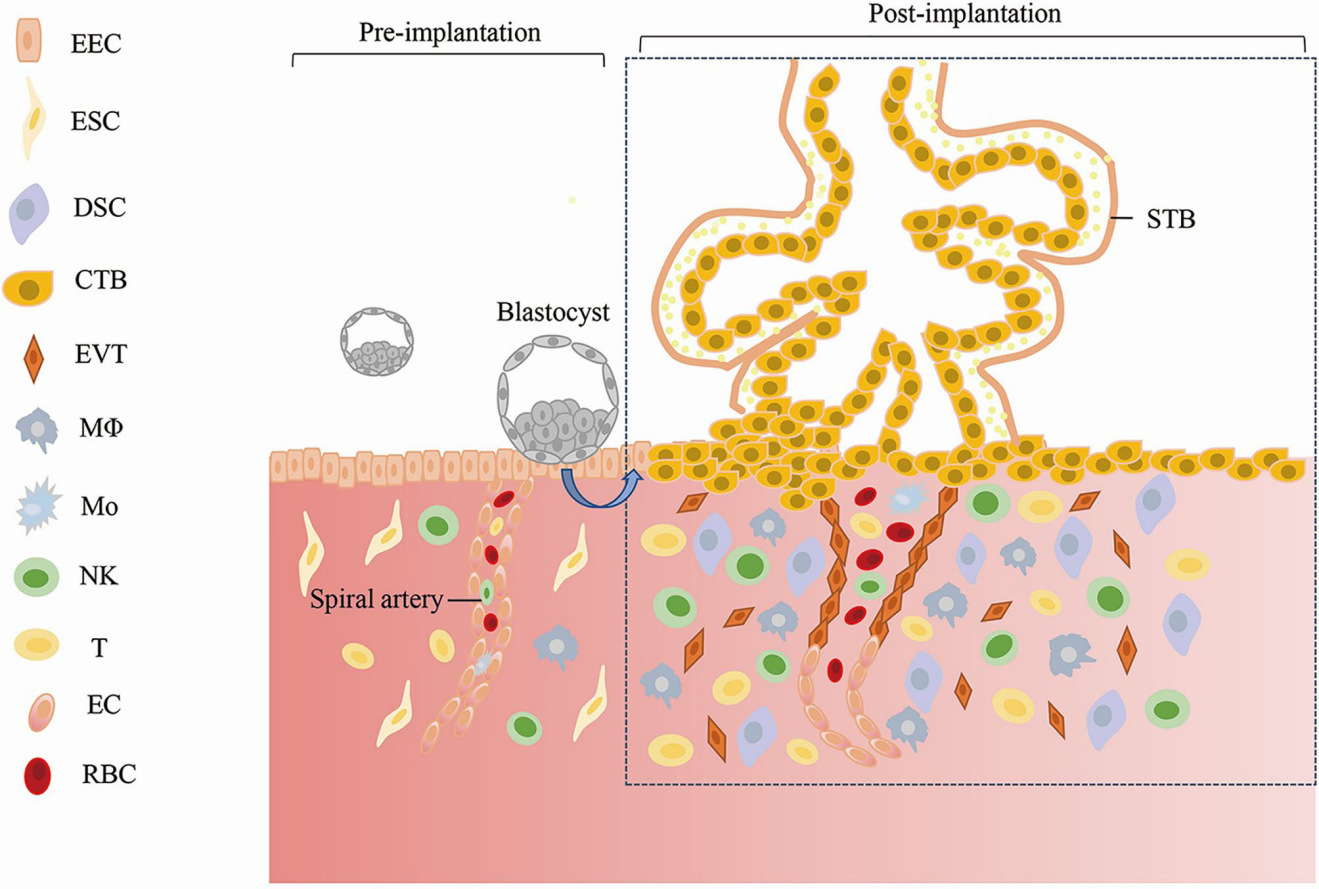
Establishment and Maintenance of Early Pregnancy. EEC, Endometrial Epithelial; Cell ESC, Endometrial Stromal Cell; DSC, Decidual Stromal Cell; CTB, Cytotrophoblast; STB, Syncytiotrophoblast; EVT, Extravillous Trophoblast; MΦ, Macrophages; Mo, Monocyte; NK, Natural Killer Cell; EC, Endothelial Cell; RBC, Red Blood Cell.

A normal pregnancy starts with the successful implantation of a promising embryo into the receptive endometrium (25). In preparation for implantation, the endometrium undergoes remodeling, which is regulated by estrogen and progesterone. During this timepoint, the endometrial epithelium becomes permissive to the adhesion of embryonic trophectoderm cells (the outer cells of the blastocyst), and then, the embryo implants into the uterus (26). After embryo implantation, the endometrium is gradually decidualized under the stimulation of ovarian hormones and other inducing factors. Decidualization is the differentiation of endometrial stromal cells into secretory decidual stromal cells. This process involves balancing pro- and anti- inflammatory cytokines (27, 28), angiogenesis, and uterine spiral artery remodeling (29). Meanwhile, the aggregation of immune cells (including uterine natural killer cells, macrophages, T cells, and dendritic cells) in the endometrium is involved in regulating the microenvironment that sustains pregnancy (30, 31). Placenta formation is the key process responsible for maintaining the growth and development of the embryo. During this process, the trophectoderm cells of the blastocyst differentiate into the extravillous trophoblast and the villous trophoblast (comprising the cytotrophoblast and syncytiotrophoblast), which form the major cell lineages of the placenta. Cytotrophoblasts can further differentiate into invasive extravillous trophoblasts (32). The proliferation and invasion of extravillous trophoblast cells into the decidua and the uterine myometrium are necessary for uterine spiral artery remodeling and establishing maternal-fetal circulation (33). The villous trophoblast contacts the maternal blood directly, which affects the material exchange among the mother, placenta and fetus (34). Extravillous trophoblast invasion and placental development are also controlled by decidualization. The establishment and maintenance of early pregnancy are represented in **Figure 1**.

## Omics Studies Investigating the Establishment and Maintenance of Early Pregnancy

During early pregnancy, extravillous trophoblasts move upstream along the arterial wall and migrate to, invade, and replace vascular smooth muscle cells and endothelial cells, thereby remodeling the uterine spiral arteries (35). Several cytokines produced by the placenta, such as vascular endothelial growth factor-A (*VEGF-A*), insulin-like growth factor, Krüppel-like factor 17, atrial natriuretic peptide may play important roles in the formation of the maternal-fetal vasculature (35, 36). Amin et al. found that the heterozygous genotype GA was significantly associated with the overexpression and underexpression of *VEGF* mRNA, while the homozygous variant genotype AA only decreased the *VEGF* mRNA levels in RPL patients by genotyping and quantitative real-time PCR (qRT–PCR-PCR) (37). Another similar study indicated that the 3’-untranslated region of *VEGF* resulted in susceptibility to RPL in Korean women (38). A recent study applying RNA sequencing of spontaneously hypertensive stroke-prone rats (SHRSP) found that the gene expression pattern of pregnant SHRSP uterine arteries was dominated by increased reactive oxygen species and downstream effectors of the renin-angiotensin-aldosterone system. The disrupted pathway involved may contribute to adverse vascular remodeling and the resultant placental ischemia and systemic vascular dysfunction (39). Uterine spiral artery remodeling is essential for promoting blood flow to the placenta and fetal development, and omics could help us better understand this process.

Successful decidualization is necessary for a normal pregnancy. By using DNA microarray and cytological verification, Lucas et al. demonstrated that in RPL, the loss of an epigenetic signature was related to the reduced expression of endometrial *HMGB2* [associated with replicative senescence of human fibroblasts (40)], and then perturbed decidualization (41). Recently, scRNA-seq data of highly proliferative mesenchymal cells (hPMCs) in the midluteal human endometrium indicated that hPMC depletion was relevant to RPL. Vascular transmigration- and decidualization -related genes, including interleukin 1 receptor like 1 (42) and prolactin, were highly expressed in hPMC (43). hPMCs play an integral role in decidualization in pregnancy (44). A proteomic analysis also confirmed that decidualization in RPL patients differed from that in normal pregnant women. Dhaenens et al. identified 1416 differentially expressed proteins (DEPs), revealing the higher expression of serotransferrin in RPL samples compared with those in normal fertile samples (45). Another study by Harden et al. showed significant differences in endometrial metabolic profiles between decidualized and nondecidualized endometrium, which may be essential for successful embryo implantation (46).

The imbalance of immune tolerance at the maternal-fetal interface is an important factor for the occurrence of RPL (47). The immunologic events occurring at the maternal-fetal interface in early pregnancy are extremely complex and involve numerous immune cells and molecules with immunoregulatory properties (47). A genome-wide transcriptome profiling of splenic B cells in pregnant and nonpregnant mice found revealed 625 upregulated and 511 downregulated transcripts in B cells from pregnant mice compared with nonpregnant mice, suggesting that B cells acquire a state of hypo-responsiveness during gestation (48). Researchers performing single-cell transcriptomic profiling of decidual tissue revealed a dramatic difference in immune cell subsets and molecular properties in RPL cases (49). In RPL patients, a decidual NK (dNK) subset that supports embryonic growth was diminished in proportion, while the ratio of another dNK subset with cytotoxic and immune-active signatures (such as pro-inflammatory CD56^+^CD16^+^ dNK subset) was significantly increased (50). Chen et al. found that a subpopulation of CSF1^+^CD59^+^ KIR-expressing dNK cells was decreased in URPL decidua (51). Treg cells constitute another important immune cell type at the maternal-fetal interface, and the transcriptional and protein expression profiles of endometrial Tregs in RPL differ from those in normal pregnant women (52).

**Figure 2:**
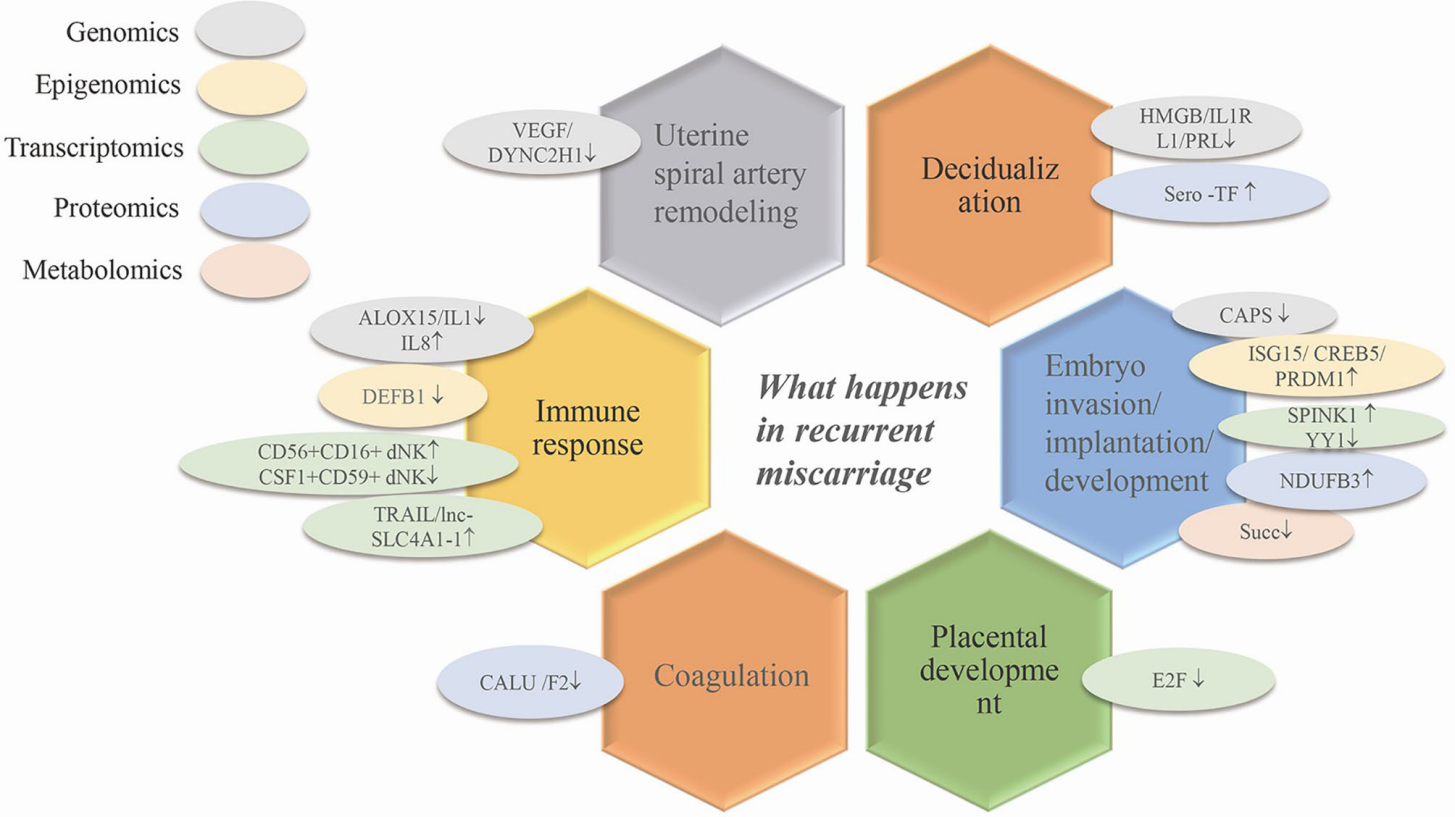
The application of omics techniques in recurrent miscarriage. Ovals of different colors represent different omics studies. VEGF, vascular endothelial growth factor; HMGB, high mobility group box; IL1RL1, interleukin 1 receptor-like 1; PRL, prolactin; ISG15, interferon stimulated gene 15; SPINK1, serine peptidase inhibitor Kazal-type 1; CREB5, cAMP responsive element binding protein5; CAPS, calcyphsine; PRDM1, positive regulatory domain 1; YY1, yin and yang 1 transcription factor; NDUFB3, NADH dehydrogenase (ubiquinone) 1 beta subcomplex 3; E2F, one transcription factor family; IL1, interleukin 1; IL8, IL1, interleukin 1; CD56+CD16+dNK/CSF1+CD59+dNK, transcriptomics *via* single cell sequencing; TRAIL, TNF-related apoptosis-inducing ligand; lnc-SLC4A1-1, solute carrier family 4, anion exchanger, member 1; DEFB1, defensin beta 1; Succ, succinate; CALU, calumenin; F2, coagulation factor II.

During early pregnancy, the changes in different genes, RNAs, proteins and metabolites associated with vascular remodeling, the abnormal expression of decidualized genes and phenotypic changes in various uterine immune cells all increase the possibility of the occurrence of RPL by affecting embryo implantation and blastocyst development (**Figure 2**). Although the pathogenesis of RPL is known to some extent, specific diagnostic biomarkers and candidate regulatory targets of RPL have not yet been identified. Thus, researchers have conducted various omics studies using decidual tissue, villi tissue, and blood from patients with RPL.

## Omics Studies of Decidua in Recurrent Pregnancy Loss

It is of great significance to study the growth, degradation and functional regulation of maternal decidual tissue to clarify the mechanism of embryo implantation and fertility regulation. Epigenomics has verified that RPL is associated with abnormal DNA modification in decidual cells. In an animal model, the abnormal methylation of the decidua has been demonstrated to be associated with pregnancy failure (53). Li et al. conducted a genome-wide screening of DNA methylation in decidual samples from women with RPL (54). The findings showed that the differentially methylated genes (*PRDM16*, *HLA-E*, *HLA-G*, and *ISG15*) were closely related to embryonic development (55–57). QRT-PCR verification showed that the mRNA expression levels of *ISG15*, *HLA-E*, and *HLA-G* were increased in RPL. Li et al. also verified the upregulated expression of *ISG15* in RPL by utilizing RNA-seq technology, and these authors found that *ISG15* was involved in the type I interferon signaling pathway by Gene Ontology (GO) and Kyoto Encyclopedia of Genes and Genomes (KEGG) pathway enrichment analyses (54). *CREB5* regulates cell growth, proliferation, differentiation and the cell cycle and belongs to the cAMP response element (*CRE*)-binding protein family (58). The upregulation of *CREB5* has been shown to promote the invasion of tumor cells (59). Yu et al. conducted an analysis of DNA methylation and gene expression, and found that hypomethylation in the *GREB5* promoter regions upregulated the mRNA and protein expression levels of *CREB5* in RPL (60). These authors further proved that *CREB5* increased migration and apoptosis in the HTR8-S/Vneo (human chorionic trophoblast cell line *in vitro*) and JEG-3 (human choriocarcinoma cell) cell lines and prolonged the cell cycle (61). Some studies have shown that *CREB5* expression and methylation are related to the plasma interleukin-6 levels. Moreover, reduced *CREB5* expression in monocytes could cause immunosuppression by increasing the tumor necrosis factor alpha (TNF-α) levels and decreasing the interleukin-10 levels in plasma (62, 63). However, the immune regulation of *CREB5* at the maternal-fetal interface and its contribution to RPL require further verification.

The upregulation of matrix metallopeptidase 26 and the serine peptidase inhibitor Kazal-type 1 (64) are crucial for trophoblastic invasion by regulating the degradation of the extracellular matrix (65). Furthermore, Krieg et al. found that all genes in the interleukin-1 pathway (downregulated) and interleukin-8 pathway (upregulated) were differentially expressed (64), suggesting that the dysregulation of genes related to immunity might contribute to RPL. Transcriptomic data also suggest that the alteration of noncoding RNA (ncRNA) expression profiles in the decidua is related to RPL. NcRNAs refer to RNAs that do not have the potential to encode proteins, including microRNAs (miRNAs), long noncoding RNAs (lncRNAs) and circular RNAs (circRNAs). NcRNAs have complex types and functions that regulate cell activity by controlling gene expression, transcription, and translation processes (66). In the decidua of RPL patients, GO and KEGG pathway analyses suggest that differentially expressed miRNAs are involved in the ErbB signaling pathway and p53 signaling pathway. LncRNAs are involved in the peroxisome proliferator-activated receptor pathway. These signaling pathways participate in the progression of RPL (67, 68). However, the function of ncRNAs in decidual cells has not been further verified. Transcriptomics data have shown the abnormal expression of genes related to embryo invasion, implantation, and development, and immune responses of decidual cells. RPL may be the result of the coregulation of these differentially expressed genes (DEGs). Unfortunately, the regulatory mechanisms of most genes or regulatory factors have not been further explored. In addition, the function of some DEGs in the decidua was further verified in HTR8-S/Vneo and JEG-3 cell lines rather than decidual cells, which is less rigorous.

Proteomics studies investigating decidua also revealed numerous DEPs and pathways involved in the pathogenesis of RPL. The complex I subunit NDUFB3 was involved in mitochondrial respiratory chain function (69), which was significantly increased in the decidua of RPL. The overexpression of NDUFB3 induced oxidative stress and apoptosis in decidual stromal cells by participating in the process of oxidative phosphorylation and affecting mitochondrial function (70). Xiong et al. suggested that 50 DEPs in the decidua were important for embryonic development and revealed that angiotensinogen (AGT) was the most important upstream regulator (71). A proteomic analysis suggested changes in protein levels related to decidual cell growth and embryonic development in decidual tissue of RPL patients, identified more biomarkers of RPL, and helped us better understand the pathogenesis of RPL.

Metabolomics provides an opportunity to study the metabolic state of tissues. Metabolic abnormalities in the decidua could result in immune dysfunction and nutritional disorders, leading to pregnancy complications, including RPL. A recent study found differentially expressed metabolites, including lipids, amino acids and organic acids, in the decidua of RPL (72), indicatingabnormalities in glucose, lipid and amino acid metabolism in decidual cells. Metabolomics studies of RPL decidua are currently limited. However, differentially expressed metabolites in patients with RPL and subsequent functional analyses could be helpful for identifying biomarkers of RPL. Omics studies investigating decidual tissue in RPL are shown in **Table 1**.

**Table 1:** Omics studies on decidual tissue of recurrent pregnancy loss patients and controls.

Reference(s)	Cell model	Omics strategy	Gestational age	Samples size	Main findings
Li et al., 2020 (54)	Decidua-	Epigenomics (DNA methylation chip)	6 to 12 weeks	RM vs NP 15 vs 15	↑ *ISG15*, *ABR*, *HLA-E*, *HLA-G*
Yu et al., 2018 (60)	Decidua	Epigenomics(DNA methylation chip)	7.715 ± 0.572 weeks	RPL vs NP 20 vs 20	↑ *CREB5*, *RBM24*, *IRF4*, *DPYSL4*
Wang et al., 2016 (67)	Decidua and villus	Transcriptomics (Small RNA deep-sequencing)	8.33 ± 1.80 weeks	RM vs NP 18 vs 15	↑ In decidua: *hsa-mir-516a-5p*, *-517a-3p*, *-519a-3p* and *-519d*
					↑ In villus: *hsa-mir-100* and *-146a-5p*
					↓ In villus: *hsa-mir-1* and *-372*
Li et al., 2021 (73)	Decidua	Transcriptomics (RNA sequencing)	28 to 82 days	RPL vs NP 15 vs 12	↑ *IFI27*, *ISG15*, *MX1*, *TNFRSF21*
Krieg et al., 2012 (64)	Decidua	Transcriptomics (RNA sequencing)	7 to 11 weeks	RPL vs NP 10 vs 6	↑ *MMP-26*, *SPINK1*, *IL8*, *IL17*, *SCGB2A1*, *HLA-DRB5*
					↓ *ZNF*
Huang et al., 2021 (68)	Decidua	Transcriptomics (RNA sequencing)	First trimester	URPL vs NP 50 vs 50	↑ *Lnc-CES1-1*
Dhaenens et al., 2019 (45)	Decidua	Proteomics (LC-HDMS)	6 to 12 weeks	RPL vs NP 3 vs 4	↓ TF
Yin et al., 2021 (70)	Decidua	Proteomics (iTRAQ technology, LC-MS/MS)	57.25 ± 9.16 days	RPL vs NP 6 vs 6	↑ COX-2, NDUFB3
Wang et al., 2021 (72)	Decidua	Metabolomics (LC-ESI-MS/MS system)	63.83 ± 7.09 days	RPL vs NP 23 vs 30	↑ l-citrulline, SM
					↓ NAAGA, CAR, PC, PE, PS, PG, LPC, LPE

RM, recurrent miscarriage; RPL, recurrent pregnancy loss; URPL, unexplained recurrent pregnancy loss; TA, elective terminations; NP, normal pregnancy; PI, primary infertility; ABR, activator of RhoGEF and GTPase; HLA-E, major histocompatibility complex, class I, E; HLA-G, major histocompatibility complex, class I, G; RBM24, RNA binding motif protein 24; IRF4, interferon regulatory factor 4; DPYSL4, dihydropyrimidinase like 4; IFI27, interferon alpha inducible protein 27; MX1, myxovirus resistance 1; TNFRSF21, tumor necrosis factor receptor superfamily, member 21; MMP-26, matrix metallopeptidase 26; SCGB2A1, secretoglobin family 2A member 1; HLA-DRB5, major histocompatibility complex, class II, DR beta 5; RPS25, 40S ribosomal protein S25; ACADVL, very long-chain specific acyl-CoA dehydrogenase; TF, serotransferrin; COX2, cytochrome c oxidase subunit 1; SM, sphingomyelin; NAAGA, N-acetylaspartylglutamic acid; CAR, carnitine; PC, phosphatidylcholine; PE, phosphatidylethanolamine; PS, phosphatidylserine; PG, phosphatidylglycerol; LPC, lysophosphatidylcholine; LPE, lysophosphatidylethanolamine.

↑, upregulation; ↓, downregulation.

## Omics Studies of Villus in Recurrent Pregnancy Loss

Placental trophoblast cells can promote embryo implantation, uterine spiral artery remodeling and placentation *via* their proliferation, invasion, and migration. Furthermore, these cells secrete numerous active substances to regulate maternal-fetal interactions to ensure the normal growth and development of the embryo and fetus (17). It is important to understand the changes in trophoblast cell function in RPL to explain its pathogenesis. Whole-exome sequencing of villi indicate that different pathogenic genes played a vital role in RPL. For example, one patient was found to have compound heterozygous mutations in dynein cytoplasmic 2 heavy chain 1, while another patient exhibited compound heterozygous variants in arachidonate 15-lipoxygenase (74). Mutations in these genes may be individually or jointly involved in the occurrence of RPL by affecting inflammation, the oxidative stress response and angiogenesis (75, 76) at the maternal-fetal interface.,

Changes in DNA methylation affect the levels of gene expression. Positive regulatory domain 1 (*PRDM1*) is a transcription inhibitor that plays a role in embryonic development (95). In RPL, hypomethylation near the transcription start site of *PRDM1* can upregulate the expression of *PRDM1*, leading to increased apoptosis and migration of trophoblast cells (78). *PRDM1* hypomethylation plays a regulatory role by recruiting transcription factors, such as Forkhead boxA1 and GATA binding protein 2, and then regulating the differentiation and development of trophoblast cells (96). Another study found that in placental villus tissue of RPL patients, the β-defensinb1 gene involved in the innate immune response had decreased methylation at the promoter region (77, 97).

Transcriptomics is widely used in the study of villi tissue of RPL. RNA sequencing results showed that Yin Yang 1 (*YY1*) mRNA expression was reduced in the trophoblasts of RPL (83). *YY1* is a transcription factor involved in embryogenesis. *YY1* can enhance the invasion and proliferation of trophoblasts by directly binding the promoter region of the matrix metalloproteinase 2 gene, which is involved in extracellular matrix remodeling during trophoblast invasion (83, 98, 99). The mRNA expression levels of placental TNF-related apoptosis-inducing ligand and S100 calcium binding protein A8 were confirmed to be elevated in RPL (84). Both genes are associated with cell apoptosis and the immune response (100, 101), and their increased expression levels indicate fetal rejection and abnormalities in trophoblasts. Another study found that most of DEGs in trophoblastic cells of RPL could bind the transcription Factor E2F (82). E2F plays an important role in maintaining trophoblastic cell function and placental development, and the absence of E2F causes disruption in the placental transcriptional network and leads to fetal death (102). Other studies have focused on the role of ncRNAs in RPL, and the differentially expressed ncRNAs participate in biological pathways, including immunity, apoptosis and hormonal regulation (79, 80). Huang et al. identified increased *lnc-SLC4A1-1* expression in URPL patients, which activated interleukin-8 (IL-8), and then exacerbated the inflammatory response of trophoblastic cells by enhancing the release of TNF-α and IL-1β (81).Transcriptome studies have revealed that the altered expression of genes or regulatory factors was associated with trophoblast proliferation, invasion, migration, and apoptosis, resulting in placental dysfunction and embryo failure to survive. In addition, the function of trophoblasts during pregnancy is affected by the immune response at the maternal-fetal interface. DEGs involved in the balance between pro- and anti-inflammatory responses also contribute to the development of RPL.

Recently, researchers have attempted to use proteomics technology to screen proteins associated with RPL in villous tissue. Compared with the normal condition, numerous DEPs in RPL, including AGT, mitogen-activated protein kinase14 (MAPK14) and Prothrombin (F2), were associated with early embryonic development (85). Among these proteins, AGT belongs to the renin-angiotensin system, MAPK14 participates in MAPK pathways, and defects in F2 lead to susceptibility to thrombosis. All these biological pathways play a role in RPL (103–105). Proteins related to regulating the function of endothelial cells and coagulation, including calumenin (CALU) and enolase 1 (ENO1), were also differentially expressed in villi (86). Defects in CALU promote coagulation and thrombosis, and a lack of ENO1 renders the placenta intolerant to hypoxia, eventually resulting in RPL (106, 107).

Research concerning the metabolomics of villous tissue in RPL is limited. A recent study showed that low succinate accumulation in villi participated in the occurrence of RPL by reducing the invasion and migration of trophoblastic cells (87). Omics of the villi of RPL patients indicated new biomarkers for the diagnosis and treatment of RPL; however, further research is needed to reveal how these differential molecules work and how they are regulated. Omics studies investigating villous tissue in RPL are shown in **Table 2**.

**Table 2:** Omics studies on villous tissue of recurrent pregnancy loss patients and controls.

Reference(s)	Cell model	Omics strategy	Gestational age	Samples size	Main findings
Qiao et al., 2016 (74)	Villus	Genomics (Whole exome sequencing)	First trimester	RPL vs NP 7 vs 2	Compound heterozygous mutations in *DYNC2H1* and *ALOX15*
Hanna et al., 2013 (77)	Villus	Epigenomics(DNA methylation chip)	9.5 ± 2.4 weeks	RM vs TA 33 vs 16	↑ *CYP1A2, AXL, H19/IGF2*, *ICR1*
					↓*DEFB1* (marginally)
Du et al., 2019 (78)	Villus	Epigenomics, Transcriptomics (DNA methylation chip, RNA sequencing)	7.826 ± 0.630 weeks	RPL vs NP 27 vs 25	↑ *PRDM1*
Wang et al., 2016 (79)	Villus	Transcriptomics (lncRNA array)	3 to 6 weeks	RPL vs NP 5 vs 5	↑ *SCARNA9*, *DIO3OS*, *H2AZ2-DT*, *RP11-379F4.4*
					↓ *PRINS*, *BMP1*, *TCL6*, *CTA833B7.2*, *RPINS*
Tang et al., 2015 (80)	Villus	Transcriptomics (miRNA microarray)	3 to 6 weeks	RPL vs NP 15 vs 15	↑ *MiR-149-3p*, *miR-4417, miR-4497* and *miR-3651*
					↓ *MiR-181d*, *miR-29b-1-5p*, *miR-24-1-5p*
Huang et al., 2018 (81)	Villus	Transcriptomics (RNA sequencing)	9.83 ± 1.25 weeks	URPL vs NP 50 vs 50	↑ *Lnc-ERGIC1-4*, *lnc-MRPS30-5*, *lnc-RCAN1-1*, *lnc-SLC4A1-1*, *lnc-TMEM135-8*, *lnc-CES1-1*
					↓ *Lnc-FGGY-4*, *lncPBK-2*, *lnc-SOX4-1*, *lncAC106873.4.1-8*
Sõber et al., 2016 (82)	Villus	Transcriptomics (RNA sequencing)	44 to 67 days	RPL vs NP 2 vs 8	↑ *ATF4*, *C3*, *PHLDA2*, *GPX4*, *ICAM1*, *SLC16A2*
					↓ *HIST1H1B*, *HIST1H4A*
Tian et al., 2016 (83)	Villus	Transcriptomics (mRNA microarray)	6 to 12 weeks	RPL vs NP 31 vs 36	↑ *CDC20*, *CTSF*, *CCR7*, *NUF2*
					↓ *IGFBP1*, *YY1*, *FGF7*, *CCNA2*
Rull et al., 2012 (84)	Villus	Transcriptomics (RNA microarray)	67.7 ± 6.6 days	RPL vs NP 13 vs 23	↑ *S100A8*, *TRAIL*
Pan et al., 2017 (85)	Villus	Proteomics (iTRAQ labeling, LC-ESI-MS/MS)	6 to 10 weeks	RPL vs NP 4 vs 4	↑ AGT, APOC1, SLC1A3, GOLT1B, PRELP
					↓ REEP6, DNTTIP2, NOLC1, SEC11C, SRSF3
Gharesi et al., 2014 (86)	Villus	Proteomics (2D-PAGE, MALDI TOF/TOF technique)	14.8 ± 2.6 weeks	RPL vs NP 5 vs 5	↓ CALU andENO1
					↑CTSD, TUBB, TUBA1, GST, PHB, ACTB
Wang et al., 2021 (87)	Decidua and villus	Metabolomics (Nuclear magnetic resonance)	First trimester	RPL vs NP 30 vs 30	↓ Succinate
					↑ SDHB

DYNC2H1, dynein cytoplasmic 2 heavy chain 1; ALOX15, arachidonate 15-lipoxygenase; CYP1A2, cytochrome P450 family 1 subfamily A member 2; AXL, receptor tyrosine kinase, H19, imprinted maternally expressed transcript; IGF2, insulin like growth factor 2; DEFB1, defensin beta 1; SCARNA9, small Cajal body-specific RNA 9; DIO3OS, opposite strand upstream RNA; PRINS, psoriasis associated non-protein coding RNA induced by stress; BMP1, bone morphogenetic protein 1; TCL6, T cell leukemia/lymphoma 6; PRINS, psoriasis associated non-protein coding RNA induced by stress; ATF4, activating transcription factor 4; C3, component 3, PHLDA2, pleckstrin homology like domain family A member 2; GPX4, glutathione peroxidase 4; ICAM1, intercellular adhesion molecule 1; SLC16A2, solute carrier family 16 member 2; HIST1H, H1.5 linker histone, cluster member; HIST1H4A, H4 clustered histone 1; CDC20, cell division cycle 20; CTSF, cathepsin F; CCR7, C-C motif chemokine receptor 7; NUF2, NUF2 component of NDC80 kinetochore complex; IGFBP1, insulin like growth factor binding protein 1; FGF7, fibroblast growth factor 7; CCNA2, cyclin A2; S100A8, S100 calcium binding protein A8; APOC1, apolipoprotein C1; SLC1A3, solute carrier family 1 member 3; GOLT1B,golgi transport 1B; PRELP, proline and arginine rich end leucine rich repeat protein; REEP6, receptor accessory protein 6; DNTTIP2, deoxynucleotidyltransferase terminal interacting protein 2; NOLC1, nucleolar and coiled-body phosphoprotein 1; SEC11C, homolog C, signal peptidase complex subunit; SRSF3, serine and arginine rich splicing factor 3; CTSD, cathepsin D; TUBB, tubulin beta; TUBA1, tubulin alpha 1; GST, glutathione S-transferas; PHB, prohibitin; ACTB, actin beta; SDHB, succinate dehydrogenase B. ↑, upregulation; ↓, downregulation.

## Omics Studies of Blood in Recurrent Pregnancy Loss

To explore the mechanism of RPL, researchers have conducted omics studies using maternal blood from RPL patients to find disease-causing genes and biomarkers of RPL. Genomics data show that a homozygous frameshift mutation in calcyphosine (*CAPS*) might be the potential pathogenesis of RPL (90). *CAPS* encodes a Ca^2+^-binding protein and is involved in the crosstalk between Ca^2+^ signaling and cAMP-protein kinase A pathways, which is crucial for embryo implantation and pregnancy maintenance (108, 109). Another molecule associated with embryo adhesion, trophinin, has also been found to be mutated in RPL. Functional defects in trophinin led to failed implantation and eventually evolved into RPL (110, 111). Genomics is a good way to identify potential targets or biomarkers for the diagnosis and treatment of RPL. These mutated genes are involved in various pathways, including cell adhesion between trophoblasts and the endometrium, embryo implantation, angiogenesis, and extracellular matrix remodeling. However, the extent to which these genes play a role in RPL has not been further verified.

The study of epigenomics in the maternal blood of RPL females is limited. Only one study showed that the methylenetetrahydrofolate reductase (*MTHFR*) gene was specifically hypermethylated in RPL (91). *MTHFR* is a thrombophilic marker involved in global DNA methylation. The methylation of the *MTHFR* gene alters the transmethylation cycle and leads to other gene methylation abnormalities, ultimately contributing to the development of RPL (112).

Using a proteomics approach, researchers have found that DEPs, including CD45, pregnancy-specific glycoprotein 1, and peroxiredoxin-2, act as predictive and diagnostic biomarkers of RPL (92). These proteins affect the invasiveness of trophoblastic cells through the Fc gamma R-mediated phagocytosis pathway, and the regulation of reactive species oxygen. Furthermore, proteins, such as insulin-like growth factor-binding protein-related protein 1, dickkopf-related protein 3 and angiopoietin-2, are significantly decreased in RPL, but the mechanism by which these proteins contribute to RPL is unclear (93).

The study of metabolomics in patients with RPL is limited. The only data available show that the levels of metabolites related to the tricarboxylic acid cycle and phenylalanine metabolism in RPL females significantly differ from those in normal controls. Lactic acid was increased, while 5-methoxytryptamine was lower in RPL patients (94). Testing metabolomics in blood provides new clues for the understanding of disease. The dysfunction of glucose, lipid and amino acid metabolism in RPL can be revealed by comparing the metabolic profiles of blood in RPL with those in normal pregnant women. This disorder might cause dysfunction of the uterus and placenta and further inhibit the growth and development of the embryo. Additionally, analyzing biomarkers in blood could to some extent reflect the condition of the decidua and villi of females and provide a new perspective for explaining the abnormal function of trophoblasts and decidual cells in RPL patients. Omics studies investigating blood in RPL are shown in **Table 3**.

**Table 3:** Omics studies on blood of recurrent pregnancy loss patients and controls.

Reference(s)	Cell model	Omics strategy	Gestational age	Samples size	Results
Quintero et al., 2017 (88)	Blood leucocytes	Genomics (Whole exome sequencing)	Before 20 weeks	RPL: 49	Mutations in *MMP10* and *FGA*
Maddirevula et al., 2020 (89)	Blood	Genomics (Whole exome sequencing)	First trimester	RPL vs PI 61 vs 14	Homozygous variant in *CCDC68*, *CBX3*, *CENPH*, *PABPC1L*, *PIF1*, *PLK1*, and *REXO4*
Pan et al., 2018 (90)	Blood	Genomics (Whole exome sequencing)	First trimester	RPL vs NP 5 vs 5	The homozygous mutation in *CAPS*
Mishra et al., 2019 (91)	Blood	Epigenomics (DNA methylation chip)	Before 24 weeks	RPL vs NP 28 vs 39	↑ *MTHFR*
Cui et al., 2019 (92)	Blood	Proteomics (iTRAQ technology, LC-MS/MS)	55.80 ± 5.85 days	RPL vs NP 30 vs 30	↓ B4DTF1, PSBG-1
					↑ B4DF70
Wu et al., 2017 (93)	Blood	Proteomics (Antibody array assay)	First trimester	RPL vs NP 60 vs 20	↓ IGFBP-rp1/IGFBP-7, Dkk3, ANGPT2
Li et al., 2018 (94)	Blood	Metabolomics (GC-MS, LC-MS)	Less than 10 weeks	RPL vs NP 50 vs 51	↑ Lactic acid
					↓ 5-methoxytryptamine

MMP-10, matrix metallopeptidase 10; FGA, fibrinogen alpha chain; CCDC68, coiled-coil domain containing 68; CBX3, chromobox 3; CENPH, centromere protein H; PABPC1L, poly(A) binding protein cytoplasmic 1 like; PIF1, 5’-to-3’ DNA helicase; PLK1, polo like kinase 1; REXO4, REX4 homolog, 3’-5’ exonuclease; B4DTF1, highly similar to Pregnancy-specific beta-1-glycoprotein 9; PSBG-1, pregnancy-specific beta-1-glycoprotein 1; B4DF70, highly similar to peroxiredoxin-2; IGFBP 7, insulin-like growth factor-binding protein 7; Dkk3, dickkopf-related protein 3; ANGPT2, angiopoietin-2. ↑, upregulation; ↓, downregulation.

In this review, we attempted to show the common genes, proteins and metabolites by integrating and analyzing data from references (**Table 4**). However, due to the limited research available and the incomplete data provided in some references, the results we obtained are not optimistic. The commonly altered genes and proteins shown in **Table 4** were derived from the results of two or more studies.

**Table 4:** The Common Genes, Proteins, Metabolites Changed in recurrent pregnancy loss patients compared with normal pregnancy females.

	Decidua	Villus	Blood
The Common Genes with Differentially Methylated Regions	*C2ORF54, TMEM161B, TBX3, SKI, TAL1, GPR137B, ITGA4* (54, 60)	*CYP1A2, DEFB1, APC, AXL, CDKN1C, MEG3, PLAGL1* (77, 78)	Only One Reference (91)
The Common mRNAs	*IFI44L, IFIT1, TNFRSF21, MMP26* (64, 73)	No Common mRNAs (82–84)	No Reference
The Common Proteins	ALB, NACA4P, KRT2, FARSA, CAT, TUBA8 (45, 70)	No Common Proteins (85, 86)	ITIH4, IGFBP4 (92, 93)
The Common metabolites	Only One Reference (72)	Only One Reference (87)	Only One Reference (94)

Genomics data do not intersect and are therefore not represented in the **Table 4**. There are many types of non-coding RNAs while few related transcriptomic studies, the sequencing results cannot be intersected. C2ORF54, chromosome 2 open reading frame 54; TMEM161B, transmembrane protein 161B; TBX3, T-box transcription factor 3; SKI, v-ski avian sarcoma viral oncogene homolog; TAL1, T-cell acute lymphocytic leukemia 1; GPR137B, G protein-coupled receptor 137B; ITGA4, integrin subunit alpha 4; APC, adenomatosis polyposis coli; CDKN1C, cyclin dependent kinase inhibitor 1C; MEG3, maternally expressed 3; PLAGL1, pleiomorphic adenoma gene-like 1; IFI44L, interferon induced protein 44 like; IFIT1, interferon induced protein with tetratricopeptide repeats 1; TNFRSF21, TNF receptor superfamily member 21; MMP26, matrix metallopeptidase 26; ALB, albumin; NACA4P, putative nascent polypeptide-associated complex subunit alpha-like protein; KRT2, type II cytoskeletal 2 epidermal; FARSA, phenylalanine–tRNA ligase alpha subunit; CAT, catalase; TUBA8, tubulin alpha-8 chain; ITIH4, inter-alpha-inhibitor heavy chain 4; IGFBP4, insulin-like growth factor-binding protein 4.

## Advantages and Limitations of Omics Studies of Recurrent Pregnancy Loss

With the development of high-throughput technology, different omics technologies have been continuously improved, providing a new perspective for the mechanistic study of RPL. The genomics technique accurately identifies the disease-causing genes of RPL and discovers the types of defective genes. Epigenomics studies do not change the DNA sequence but allow us to understand the mechanism of RPL from the perspective of gene modification. Transcriptomics suggests the regulatory mechanism of RPL-related RNA. Proteomics and metabolomics provide new biomarkers and analyze the biological pathways, in which these molecules participate. By combining these omics findings, we infer that most differentially expressed molecules associated with RPL are involved in the processes of decidualization, embryo implantation, trophoblast cell differentiation, invasion and apoptosis, placental development, fetal development, the immune response, and coagulation. Omics research investigating RPL is an emerging field, and there are still some shortcomings in the related research. First, the sample sizes in most studies are small, the reproducibility of the data is poor, and it cannot be ruled out that these experimental results are caused by individual differences. Therefore, further studies with a larger sample size are needed to support the current results. Second, most studies were only performed with omics combined with a bioinformatics analysis. The results only show the potential pathways or regulation modules in which these molecules participate. To illustrate the specific mechanisms of these molecules in RPL, more basic research is needed. Third, most current studies were conducted at the single-omics level, and few studies integrated multiomics to verify the pathogenesis of RPL. Subsequent studies should perform multiple omics tests in patients with RPL, integrate information from different omics, and delve deeper into the pathogenesis of RPL. Finally, no studies performed proteomics in RPL to determine the posttranslational modifications of proteins, such as phosphorylation, ubiquitination, acetylation, and glycosylation, which can verify the biological processes more precisely. In addition, isotope labeling in differential metabolites can be used in a metabolic flux analysis to better understand the dynamics of metabolic systems in RPL. In summary, there is still a long way to go for research investigating the etiology, treatment and prevention of RPL based on high-throughput omics.

## Conclusion

The pathogenesis of RPL is complex, and related to hormonal, environmental, and genetic factors. Furthermore, the causes of approximately 50% of RPL cases are unknown, and its incidence rate is increasing. Although assisted reproductive technology can be used to promote the pregnancy rate, the success rate is not very satisfactory. RPL patients are often under tremendous physical and psychological pressure. Therefore, in future studies, it is indispensable to comprehensively analyze the pathogenesis of RPL from different biochemical and biophysical processes. As omics investigates pathomechanisms from multiple dimensions, such as DNA, RNA, proteins, and metabolites, we hope that multiomics could provide more clues regarding the diagnosis and treatment of PRL.

## Author Contributions

JL, LW and JD contributed equally to the preparation and the writing of the manuscript. YC and LD critically provided critical feedback and helped shape the manuscript. LL, YZ and TY supervised and revised the manuscript. All authors approved the final version of the manuscript for publication.

## Funding

The project was funded by National Key Research & Developmental Program of China (2018YFC1004601, 2018YFC1003900/04), Guangdong Basic and Applied Basic Research Foundation (2019A1515011315), Shenzhen Fundamental Research Program (JCYJ20190813161010761, JCYJ20210324123601004), and National Natural Science Foundation of China (No. 82071655, No. 81801540).

## Conflict of Interest

The authors declare that the research was conducted in the absence of any commercial or financial relationships that could be construed as a potential conflict of interest.

## Publisher’s Note

All claims expressed in this article are solely those of the authors and do not necessarily represent those of their affiliated organizations, or those of the publisher, the editors and the reviewers. Any product that may be evaluated in this article, or claim that may be made by its manufacturer, is not guaranteed or endorsed by the publisher.
